# A Comparative Study on the Paradoxical Relationship Between Heavy Metal Exposure and Kidney Function

**DOI:** 10.3390/diagnostics15010086

**Published:** 2025-01-02

**Authors:** Jee Hyun Rho, Seungho Lee, Jung-Yeon Kwon, Young-Seoub Hong

**Affiliations:** 1 Department of Preventive Medicine, College of Medicine, Dong-A University, Busan 49201, Republic of Korea; rogiken@dau.ac.kr (J.H.R.); kitty1004ki@dau.ac.kr (J.-Y.K.); 2 Environmental Health Center, Dong-A University, Busan 49201, Republic of Korea

**Keywords:** KoNEHS, FROM study, eGFR, heavy metals

## Abstract

**Introduction**: Korea has higher levels of heavy metals compared to other countries, raising the need to study the health impacts on vulnerable populations. This study examined the effects of heavy metal exposure—lead, mercury, and cadmium—on kidney function in residents of environmentally vulnerable areas compared to the general population in Korea. **Methods**: Epidemiological studies in vulnerable areas and official data from the Fourth Korean National Environmental Health Survey were analyzed to assess blood levels of lead and mercury and urinary cadmium. An integrated heavy metal concentration was calculated, combining the levels of these metals. Kidney function was evaluated using the estimated glomerular filtration rate (eGFR), classified into normal, mildly reduced, and impaired. Correlation and logistic regression analyses were used to examine relationships between heavy metal levels and eGFR. **Results**: The integrated heavy metal concentration in vulnerable areas was higher than in the general population. In the general population, increased heavy metal levels were associated with a decrease in eGFR, whereas in vulnerable areas, eGFR increased with higher heavy metal levels. In the general population, a rise in urinary cadmium increased the risk of eGFR decline by 19.9%, while in vulnerable areas, higher urinary cadmium reduced this risk by 23.3%. **Conclusions**: Contrasting relationships between heavy metal exposure and eGFR in vulnerable areas versus the general population may be due to long-term exposure and reduced renal excretion. This study underscores the need for continued monitoring in vulnerable areas, and future research should identify eGFR thresholds that correlate with heavy metal level shifts.

## 1. Introduction

Globally, toxic metals such as cadmium (Cd), lead (Pb), and mercury (Hg) are causing considerable environmental pollution, including air, soil, and water contamination, and pose serious threats to human health [1,2]. These toxic metals enter the body primarily through the digestive and respiratory systems, and even prolonged exposure to trace amounts can have detrimental health effects [3]. The kidneys, as a major pathway for excreting heavy metals, are particularly vulnerable to heavy metal toxicity [4]. Notably, kidney function may decline by over 50% without any noticeable symptoms, making early detection of kidney disease difficult. Once damage occurs, recovery is challenging, making kidney health an important public health issue. Compared to the prevalence of kidney disease, public awareness remains relatively low [5,6].

Pb, classified as Group 2A (probably carcinogenic to humans) by the International Agency for Research on Cancer, is known to cause neurotoxicity. Long-term Pb exposure can also increase the risk of renal proximal tubular changes, as the kidneys are a major excretory organ [7,8,9]. Pb exposure can damage various organs, including the central nervous system, lungs, liver, gastrointestinal tract, and cardiovascular system. Additionally, Pb can disrupt the oxidation–reduction balance and trigger inflammation in various organs [10]. Hg is another toxic pollutant that, even in trace amounts, can accumulate in the kidneys, causing epithelial damage and necrosis in the proximal tubules and rectum [11,12,13]. It also impacts multiple organ systems, including the central nervous, cardiovascular, respiratory, endocrine, and immune systems [10,14]. Cd accumulates in the renal cortex after low-dose chronic exposure, leading to a decreased glomerular filtration rate (GFR) and increased renal toxicity. Cd exposure is also linked to degenerative bone disease and damage to organs such as the liver, gastrointestinal tract, and lungs, and even cancer [8,15,16].

In Korea, the Korean National Environmental Health Survey (KoNEHS) monitors heavy metal exposure in the general population every 3 years. It aims to continuously assess environmental health from regional to national levels and evaluate the impact of hazardous substances on human health. Although heavy metal levels in Korean adults are trending downwards, they remain higher than levels reported in international biomonitoring surveys such as the US National Health and Nutrition Examination Survey (NHANES), the Canadian Health Measures Survey (CHMS), and the German Environmental Survey (GerES) [17,18,19]. Additionally, studies have shown that individuals living in environmentally vulnerable areas have higher heavy metal levels compared with the general population [20,21,22]. To address this, ongoing research in Korea focuses on identifying vulnerable areas and monitoring environmental hazards and their health impact on residents. Since 2021, the Forensic Research via Omics Markers (FROM) study has been analyzing environmental hazard exposure and disease biomarkers by collecting biological samples from residents of vulnerable areas. So far, 1157 participants have been recruited, and heavy metal concentration analysis has been completed.

Generally, assessments of hazardous substance exposure have focused on individual metals. However, in cases of simultaneous exposure to multiple heavy metals, reference values for individual metals may not accurately reflect the health impacts caused by combined exposure [23]. Hambach et al. [24] reported that Pb enhances the association between Cd exposure and renal biomarkers, and Sanders et al. [25] suggested that combined exposure to Cd and Pb has a strong association with a decreased eGFR. According to a 2020 study by Luo et al. [26], exposure to heavy metal mixtures, including cobalt, Cd, and Pb, can lead to declines in renal function. Moreover, simultaneous exposure to Pb and Cd was reported to be a potent determinant of chronic kidney disease [27]. Despite these findings, studies on the health impact of integrated heavy metal exposure, particularly on renal function, in residents of vulnerable areas are still limited.

This study aimed to analyze the correlation between heavy metal exposure levels and estimated glomerular filtration rate (eGFR), an indicator of renal function. Using reference data from the fourth KoNEHS to represent the general population and data from the FROM study for vulnerable areas, we calculated the levels of integrated heavy metal exposure and examined its correlation with eGFR.

## 2. Materials and Methods

### 2.1. Study Subjects

The KoNEHS, conducted by the National Institute of Environmental Research, has been carried out in a 3-year cycle since 2009, in accordance with Article 14 of the Environmental Health Act. Sample households are selected from the general population, and in vivo monitoring of environmentally hazardous substances is performed. A questionnaire survey is also used to identify the factors influencing exposure. Associations with clinical indicators are analyzed to assess early health impacts from environmental hazards. This study utilized data from the fourth KoNEHS, conducted between 2018 and 2020, involving 2988 adults aged ≥ 19 years. After excluding four participants with missing blood Pb, blood Hg, and urinary Cd values, data from 2984 adults were analyzed.

The FROM study aims to develop biomarkers for environmental hazard exposure and track disease biomarkers for assessing environmental diseases. It aims to verify, apply, and validate these biomarkers in patients affected by environmental diseases and in environmentally vulnerable areas. To select study locations, reports on vulnerable areas in Korea published between 1997 and 2021 were reviewed. Based on these data, 13 areas with high frequencies of exposure to heavy metals and organic compounds were selected for the study [28]. From June 2021, a total of 1157 adults aged ≥ 19 years, living within a 10 km radius of these vulnerable areas and who consented to participate, were recruited over 2 years. A questionnaire survey was conducted to collect demographic data (including gender and age), area of residence, and length of residence. Blood and urine samples were collected to determine in vivo exposure to hazardous substances. Details regarding the research team, study areas, and methods can be found in a previous publication [29].

The KoNEHS and the FROM study were conducted with prior written consent obtained from all subjects.

### 2.2. Biological Sample Collection and Analysis Methods

#### 2.2.1. The Fourth KoNEHS

All procedures for biological sample collection and heavy metal analysis followed the guidelines and manual provided by the fourth KoNEHS [30]. Blood samples were collected in ethylenediaminetetraacetic acid (EDTA) tubes and serum separation tubes (SST). EDTA blood samples were mixed for 30 min using a roller mixer, transported in a refrigerated state, and then aliquoted and frozen for storage. Whole-blood samples were collected in SST, mixed thoroughly by inverting the tube, and allowed to stand for 30 min. These samples were centrifuged at 3000 revolutions per minute (RPM) for 10 min to separate the serum, which was then freeze-stored until analysis. Urine samples were collected in 65 mL volumes, the minimum required for analysis, in urine collection cups. The urine samples were transported in a refrigerated, light-shielded state, aliquoted, and then freeze-stored. All samples were sent to designated laboratories for analysis according to the fourth KoNEHS protocol.

Blood Pb and urinary Cd were analyzed using a graphite furnace atomic absorption spectrometer; in contrast, blood Hg was measured at a wavelength of 253.65 mm using a direct mercury analyzer (DMA-80, Milestone, Bergamo, Italy) with gold amalgamation. Sample concentrations were determined using calibration curves from the standard addition method, where a known quantity of a standard solution is added to the sample. Values below the limit of detection (LOD) were adjusted by using LOD/√2. To ensure result reliability, certified reference materials were used for internal and external quality control (QC), confirming both accuracy and precision.

Serum creatinine was measured via colorimetry using the Jaffe reaction (alkaline picrate, kinetic with blank rate correction method, ADVIA 1800, Siemens, Berlin, Germany) by analyzing absorbance at a wavelength of 505/571 nm. QC for serum creatinine was performed using assayed liquid control levels 1 and 2 (Bio-Rad, Hayward, CA, USA), and sample concentrations within the measurable range (0.1–25.0 mg/dL) were analyzed accordingly.

#### 2.2.2. FROM Study

Methods for biological sample collection and analysis in the FROM study were detailed in a previous article [29]. Briefly, whole blood was collected in EDTA tubes, mixed for 30 min in a roller mixer, and then transported in a refrigerated state. For serum separation, whole blood was collected in an SST, mixed by inverting the tube, and left to stand for 30 min before being centrifuged at 3000 RPM for 10 min. The separated serum was freeze-stored until analysis. Urine samples were collected in spot urine cups and dispensed into 15 mL tubes, then transported in a refrigerated state and freeze-stored until analysis. Blood Pb was analyzed using an inductively coupled plasma mass spectrometer (ICP-MS; NexION 200B, PerkinElmer, Waltham, MA, USA); in contrast, urinary Cd was analyzed using an Agilent 7700x ICP-MS (Agilent Technologies, Santa Clara, CA, USA). Blood Hg was analyzed using a gold amalgamation direct mercury analyzer (MA-3000, NIC, Tokyo, Japan). Blood and urine samples were brought to room temperature and mixed in a roller mixer for at least 30 min before heavy metal analysis. Heavy metal standard solutions were prepared by stepwise dilution, and the calibration curve was validated by testing one concentration for every 20 samples. Whole Blood ClinChek levels 1 and 2 (RECIPE Chemicals, München, Germany) and Urine ClinChek levels 1 and 2 (RECIPE Chemicals, Germany) were used as QC materials to verify the accuracy of the method. Values below the LOD were treated as LOD/√2. Serum creatinine was measured using a creatinine test kit (Roche) based on the modified Jaffe method.

### 2.3. Calculation of eGFR

To calculate eGFR, a key indicator of renal function [31], the formula proposed by the Chronic Kidney Disease Epidemiology Collaboration (CKD-EPI, 2021) was used [32,33]: eGFR (mL/min/1.73 m^2^) = 142 × min (serum creatinine/k,1)^α^ × max (serum creatinine/k,1)^−1.200^ × 0.9938^age^ (for females: × 1.012), where k = 0.7 for females and 0.9 for males, and α = −0.241 for females and −0.302 for males. In this study, eGFR was classified according to international clinical practice guidelines into three stages: normal (eGFR ≥ 90 mL/min/1.73 m^2^), slightly reduced (eGFR 60–<90 mL/min/1.73 m^2^), and impaired (eGFR < 60 mL/min/1.73 m^2^) [34].

### 2.4. Statistical Analysis

Data from the fourth KoNEHS represent the general population; in contrast, data from the FROM study represent residents of vulnerable areas. Consequently, all statistical analyses were based on comparative assessments between residents of vulnerable areas (FROM) and the general population (KoNEHS). The normality of variables in both datasets was evaluated, and for variables with skewed distributions, the geometric mean (GM) and 95% confidence interval (CI) were presented. The integrated heavy metal concentration (Σ HM) was calculated by summing the concentrations of blood Pb, blood Hg, and urinary Cd. One-way analysis of variance was conducted to compare Σ HM and individual heavy metal concentrations across groups categorized by renal function, as indicated by eGFR: normal (≥90 mL/min/1.73 m^2^), slightly reduced (60–<90 mL/min/1.73 m^2^), and impaired (<60 mL/min/1.73 m^2^). To analyze the correlation between Σ HM and eGFR, Spearman’s correlation analysis was performed, excluding outliers in each area as identified by the Bland Altman plot. Next, logistic regression analysis was used to assess associations between eGFR, individual heavy metal concentrations, and Σ HM. Heavy metals were analyzed using log-transformed concentrations, with eGFR thresholds of 60 mL/min/1.73 m^2^ and 90 mL/min/1.73 m^2^ as reference points. All models were adjusted for age, gender, and BMI. Models 1, 2, and 3 were used to evaluate the effects of blood Pb, blood Hg, and urinary Cd, respectively. Model 4 was used to examine the effects of the combined concentrations of blood Pb, blood Hg, and urinary Cd. Lastly, Model 5 included Σ HM. All statistical analyses were performed using IBM SPSS version 23.0 (IBM Corp., Armonk, NY, USA), with statistical significance set at *p* < 0.05.

## 3. Results

The GM and 95% CI of heavy metal concentrations in the subjects are presented in Table 1. Analysis of the fourth KoNHES data revealed that the GM concentrations of blood Pb, blood Hg, and urinary Cd in Korean adults from the general population were 1.58 µg/dL (95% CI: 1.56, 1.61), 3.11 µg/L (95% CI: 3.04, 3.19), and 0.49 µg/L (95% CI: 0.47, 0.50), respectively. In contrast, data from the FROM study, which represents residents of vulnerable areas, showed a GM concentration of 1.76 µg/dL for blood Pb, 3.24 µg/dL for blood Hg, and 1.34 µg/dL for urinary Cd. The study areas are listed on the left side of Table 1.

The mean concentration of blood Pb was notably high in six areas (A, B, C, E, F, and K) among the environmentally vulnerable areas, with Area C showing the highest concentration (GM: 3.71 µg/dL), more than double that of the general population. In terms of blood Hg, the areas where the mean concentration exceeded that of the KoNEHS were B, E, G, H, K, and L, with Area L showing the highest concentration at 5.71 µg/L. The mean urinary Cd concentration across 13 vulnerable areas ranged from 0.81 to 2.66 µg/L, which was higher than the 0.49 µg/L observed in the general population. Area B exhibited the highest concentration, over five times that of the general population.

Table 2 presents the distribution of blood Pb, blood Hg, urinary Cd, and Σ HM for the general population and residents of vulnerable areas. The mean Σ HM for the KoNEHS was 6.53 (GM: 5.70); in contrast, the FROM study had a mean Σ HM that was approximately 25% higher, at 8.09 (GM: 7.24). In nine vulnerable areas (A, B, C, E, G, H, J, K, and L), the Σ HM was higher than that in the general population. Based on the 95th percentile concentration, five areas (A, B, C, E, and L) exhibited higher Σ HM levels than the general population in the fourth KoNEHS. However, the general population had a higher maximum Σ HM compared with the residents of vulnerable areas.

**Figure 1:**
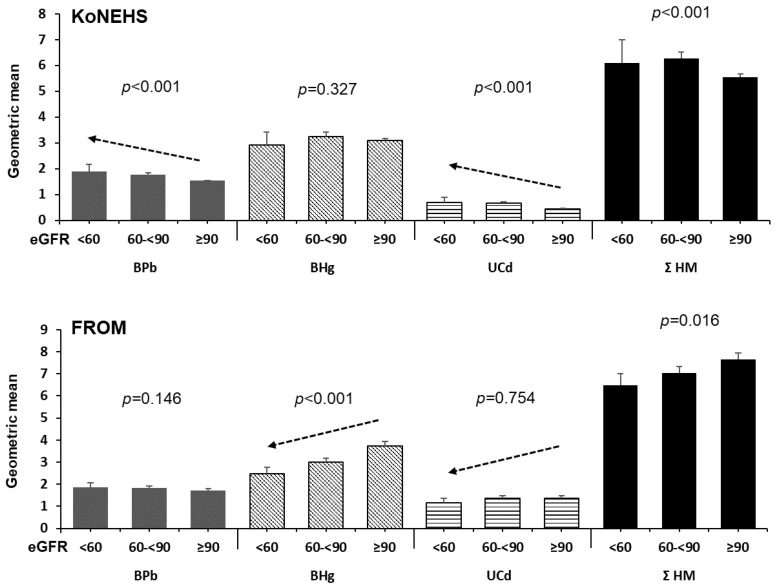
Estimated GFR levels according to the individual heavy metal concentrations and the integrated heavy metal concentrations—the general population (KoNEHS, **above**) and vulnerable areas (FROM, **below**). [Note] The *p*-value indicates the significance of differences using one-way analysis of variance (ANOVA).

The relationship between heavy metal concentrations and renal function, as indicated by eGFR, was analyzed by dividing eGFR into three stages: normal (≥90 mL/min/1.73 m^2^), slightly reduced (60–<90 mL/min/1.73 m^2^), and impaired (<60 mL/min/1.73 m^2^) (Figure 1). In the general population (KoNEHS), blood Pb, urinary Cd, and Σ HM, but not blood Hg, showed significant differences in concentrations according to eGFR stages (*p* < 0.001). Blood Pb and urinary Cd concentrations tended to increase as renal function declined. Conversely, in the FROM study, representing residents of vulnerable areas, no statistically significant differences in blood Pb or urinary Cd were observed across eGFR stages. However, blood Hg and Σ HM decreased with decreasing eGFR values, showing statistically significant differences between the groups. The number of subjects in each eGFR stage for both the KoNEHS and the FROM study is provided in Supplementary Table S1.

**Figure 2:**
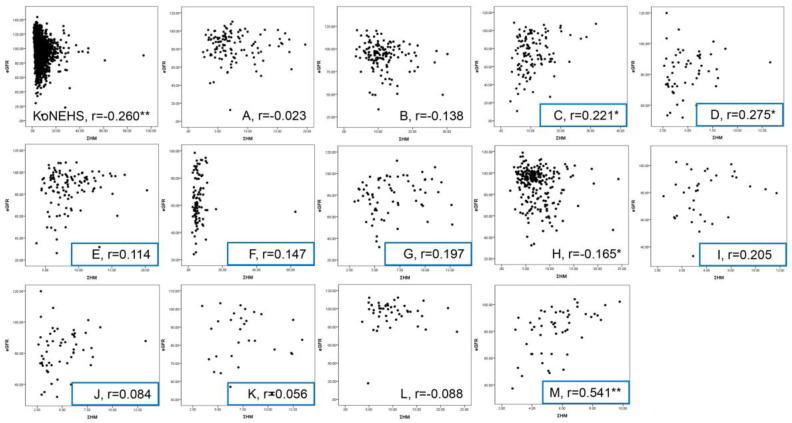
The correlation between eGFR and the integrated heavy metal concentrations in the general population (4th KoNEHS) and vulnerable areas (FROM). [Note] The correlation coefficients were derived from Spearman correlation analyses. The asterisk mark indicates statistical significance, with * representing <0.05 and ** representing <0.01. Blue box indicates the positive correlation coefficient.

Figure 2 illustrates the correlation between eGFR and Σ HM in the general population and residents of vulnerable areas. In the general population (KoNEHS), a weakly negative correlation (*r* = −0.260, *p* ≤ 0.01) was observed, indicating that eGFR tended to decrease as Σ HM increased. However, in vulnerable areas (FROM), the correlation between eGFR and Σ HM varied, showing a positive correlation in nine areas (C, D, E, F, G, I, J, K, and M), with eGFR decreasing as Σ HM decreased. Excluding Area M, the correlation coefficients were generally small (*r* = 0.13, *p* ≤ 0.001).

Logistic regression analysis was used to assess the effects of each heavy metal on eGFR. In the general population, urinary Cd made a significant contribution to reducing the eGFR, with an odds ratio (OR) of above 1 (Table 3). A one-logarithmic-unit increase in urinary Cd was associated with a 19.9% increase in the OR of eGFR reduction to below 60 mL/min/1.73 m^2^ (KoNEHS, Model 3), and the OR increased by 19.4% when adjusted for blood Pb and blood Hg (KoNEHS, Model 4). The ORs for other heavy metals and Σ HM were not significant. In the general population aged ≥ 50 years, only the OR for urinary Cd was significant (OR for urinary Cd: 1.21; adjusted OR: 1.206).

In vulnerable areas, the ORs for blood Hg and urinary Cd reducing eGFR were below 1, showing a statistically significant decrease. A one-logarithmic-unit increase in blood Hg was associated with a 45.3% decrease in the OR of eGFR reduction to below 60 mL/min/1.73 m^2^ (FROM, Model 2). Similarly, a one-logarithmic-unit increase in urinary Cd was associated with a 23.3% decrease in the OR of eGFR reduction (FROM, Model 3). In Models 4 and 5, which considered all three heavy metals and Σ HM, respectively, an increase in Σ HM was also associated with a decrease in eGFR, demonstrating a proportional relationship between heavy metal concentrations and renal function.

## 4. Discussion

This study utilized data from both the general Korean population (fourth KoNEHS) and environmentally vulnerable areas (FROM study) to explore the correlation between heavy metal concentrations and eGFR. The results showed that heavy metal levels were considerably higher in vulnerable areas compared to the general population, particularly for Pb and Cd. Blood Pb concentrations in vulnerable areas were nearly double, and Cd levels were elevated across all vulnerable regions relative to the general population. Correlation and logistic regression analyses revealed that in the general population, eGFR tended to decrease as heavy metal concentrations increased. However, in vulnerable areas, the OR for eGFR reduction decreased as heavy metal concentrations increased, indicating a proportional relationship between these metals and eGFR.

In the general population, the mean concentrations of blood Pb, blood Hg, and urinary Cd were 1.58 µg/dL, 3.11 µg/L, and 0.49 µg/L, respectively. Comparatively, in the US NHANES (2017–2018) [35], the mean blood Pb, blood Hg, and urinary Cd concentrations among Americans aged ≥ 20 years were 0.86 µg/dL, 0.75 µg/L, and 0.18 µg/L, respectively. In the Canadian CHMS (Cycle 6, ages 3–29) [36], the concentrations were 0.81 µg/dL, 0.71 µg/L, and 0.19 µg/L, respectively. These results indicate that the general population in Korea had more than four times the blood Hg levels and over twice the urinary Cd levels compared to those in the US and Canada. Moreover, blood Pb and blood Hg concentrations among participants in Germany’s GerES III (ages 18–69) [37] were significantly lower—0.44 µg/L and 0.58 µg/L, respectively—than those observed in Korea. Similarly, data from the European HBM4EU study (involving nine countries and participants 20–39 years of age) [38] also supported the higher heavy metal concentrations in Koreans compared with individuals from other countries in Europe.

Previous research has consistently shown that heavy metal levels are higher in residents of environmentally vulnerable areas than in the general population or control groups. For example, a study on individuals living near abandoned mines reported elevated concentrations of blood Pb (3.26 µg/dL), blood Hg (3.35 µg/L), and urinary Cd (1.11 µg/g creatinine) [39]. Similarly, Rho et al. [21] found that residents near coal-fired power plants had blood Pb, blood Hg, and urinary Cd levels of 1.35 µg/dL, 3.16 µg/L, and 1.58 µg/g creatinine, respectively, with urinary Cd levels more than double those of the general population. Another study found that subjects living near a zine smelter had significantly higher levels of blood Pb (3.47 ± 1.70 µg/dL) and urinary Cd (1.36 ± 2.58 µg/L) compared to controls (2.67 ± 1.39 µg/dL for blood Pb and 0.80 ± 2.60 µg/L for urinary Cd) [40]. These findings also demonstrate that heavy metal concentrations were generally higher among residents of vulnerable areas than in the general population.

Globally, numerous studies have investigated the link between heavy metal exposure and declines in eGFR. In the US, research on chronic kidney disease (CKD) risk factors revealed that individuals with high blood Cd (>2.4 µg/dL) and high blood Pb (>0.7 µg/L) had an increased OR (1.56 and 1.40 times higher, respectively) for reduced eGFR. The simultaneous exposure to these two metals has been reported as a strong determinant of the development of CKD. Similarly, adults aged ≥ 20 years with blood Cd concentrations ≥1 μg/L had a 1.48 times higher OR for CKD (*p* = 0.046) [41]. The present study’s findings align with these studies, showing that increased urinary Cd was associated with a reduced eGFR in the general population (OR: 1.199, Table 3).

However, the correlation between heavy metals and eGFR was not particularly strong. In a study by Kim et al. [42], the correlation coefficients between eGFR and Pb, Hg, and Cd were −0.19, −0.07, and −0.13, respectively. Park et al. [43] also found weak but significant negative correlations between eGFR and blood Pb (r = −0.255, *p* ≤ 0.001), Cd (r = −0.164, *p* ≤ 0.001), and nickel (r = −0.056, *p* ≤ 0.05). The present study also found a weak negative correlation (r = −0.26) between heavy metals and eGFR in the general population (KoNEHS). Interestingly, a weak positive correlation (r = 0.13) was observed among residents of vulnerable areas (FROM study).

Some studies have reported a positive correlation between an increased eGFR and higher heavy metal concentrations. Hwangbo et al. [44] found a gender difference, where eGFR increased by 1.84 mL/min/1.73 m^2^ in male adults when blood Cd levels doubled; in contrast, eGFR decreased in female adults with increased blood Cd. Jain [45] reported that both blood and urinary Cd levels varied according to the stage of glomerular function, increasing up to an eGFR of 45–60 mL/min/1.73 m^2^ but leveling off after an eGFR of 15–45 mL/min/1.73 m^2^, suggesting a new steady state in renal failure. Additionally, another study found negative associations between eGFR and increased blood Cd and Pb levels; in contrast, positive associations were observed with increased urinary Cd and Pb levels (β: 3.55 for urinary Cd and 8.51 for urinary Pb) [46]. The authors attributed this finding to the hypothesis that impaired renal function reduces Cd excretion in urine, leading to higher blood Cd levels [46]. These results align with the pattern observed in the present study, which found an increase in eGFR with rising heavy metal concentrations.

In this study, a large proportion of participants from vulnerable areas were older adults (aged ≥ 60 years), many of whom had reduced eGFR due to chronic heavy metal exposure (Supplementary Table S1). Aging kidneys can undergo microstructural changes such as glomerulosclerosis and tubulointerstitial fibrosis, which can compromise GFR [47]. Aaseth et al. [48] reported that older individuals may also accumulate higher levels of metal toxicants due to physiological changes and decreased renal function. Denic et al. [49] expressed concerns that using a fixed GFR value (<60 mL/min/1.73 m^2^) to define chronic diseases in older adults may lead to overdiagnosis. When eGFR declines, urinary excretion of Cd decreases, potentially increasing circulating blood levels of heavy metals [50,51].

A study examining the combined exposure to heavy metals in older American adults aged 60 years and older found a positive correlation between combined heavy metal exposure and eGFR and a negative correlation with CKD [52]. Johri et al. [53] highlighted the importance of evaluating combined exposure to heavy metals when assessing renal function, as the effects can be either synergistic or antagonistic. Similarly, Weaver et al. [54] reported that urine Cd and thallium levels were significantly positively associated with eGFR (mL/min/1.73 m^2^) when adjusted for urine creatinine (β = 3.1 for Cd and 3.6 for Thallium). Previous research has shown that Pb and Cd, which accumulate in the kidneys, are excreted through them, and long-term exposure to these heavy metals can impair renal function and lead to various kidney diseases [16,55]. The nephrotoxic effects of combined heavy metal exposure may result from synergistic interactions, leading to renal tubular damage, epithelial cell necrosis, a decreased eGFR, oxidative stress-induced cell death, and structural damage to the kidneys [8,10,56]. Tsai et al. [57] reported that the ORs for a decreased eGFR in individuals with higher blood Pb and urinary copper (Cu) levels were 3.727 (*p* = 0.022) and 1.163 (*p* = 0.009), respectively. They also noted that simultaneous exposure to Cd, Cu, Pb, and chromium may exhibit synergistic effects, increasing the risk of proteinuria. This suggests that combined exposure to multiple heavy metals carries a higher risk of a decreased eGFR compared to exposure to Cd alone.

The current study utilized data from the fourth KoNEHS, representing the general population in Korea, along with data from a large-scale bio-monitoring study of residents in environmentally vulnerable areas, to assess heavy metal exposure and its correlation with GFR across different levels of exposure. However, there are limitations to this study. First, as a cross-sectional survey, it could not fully capture the long-term exposure levels of residents in environmentally vulnerable areas. Additionally, it did not account for various other factors beyond proximity to vulnerable facilities. Second, this study did not consider the impact of other heavy metals not included in the fourth KoNEHS or other markers of renal function, such as β2-microglobulin and N-acetyl-β-D-glycosaminidase.

Despite these limitations, this study is significant for its focus on vulnerable areas in Korea, conducting bio-monitoring to assess the exposure of residents relative to the general population. The results indicate that an increase in heavy metal concentrations was associated with a decrease in eGFR in the general population and revealed a proportional relationship between eGFR and urinary heavy metal concentrations, particularly Cd, in the stages of eGFR decline. Many studies have also reported that increasing heavy metal levels are linked to a decreased eGFR [50,58], with age adjusted as a covariate in the analyses. Therefore, examining the effects of age-related heavy metal accumulation on eGFR levels represents a novel research topic that warrants further investigation. Additionally, determining the threshold at which the direction of the correlation between heavy metals and eGFR changes across stages of eGFR decline will be a goal for future research.

## 5. Conclusions

In this study, data from the fourth KoNEHS, representing the general population in Korea, and the FROM study, representing environmentally vulnerable areas, were analyzed to examine the correlation between heavy metal concentrations from bio-monitoring and eGFR. First, in environmentally vulnerable areas compared with the general population, Pb levels were approximately twice as high, and Cd levels were elevated in all regions. The correlation and logistic regression analysis results showed that increased heavy metal concentrations were associated with a decreased eGFR in the general population. However, in environmentally vulnerable areas, higher heavy metal concentrations were correlated with a reduced OR for a decline in eGFR, indicating a proportional relationship between heavy metal concentrations and eGFR. Although this study focused on exposure to three major heavy metals, future studies should assess the health impacts of combined exposure to various other heavy metals. Given that residents of environmentally vulnerable areas had higher heavy metal concentrations than the general population, ongoing monitoring and management of chronic diseases in these regions are essential.

## Figures and Tables

**Table 1:** The distribution of heavy metal concentrations in the general population (4th KoNEHS) and environmentally vulnerable areas (FROM).

				GM (95% CI)		
			N	Blood Pb (μg/dL)	Blood Hg (μg/L)	Urine Cd (μg/L)
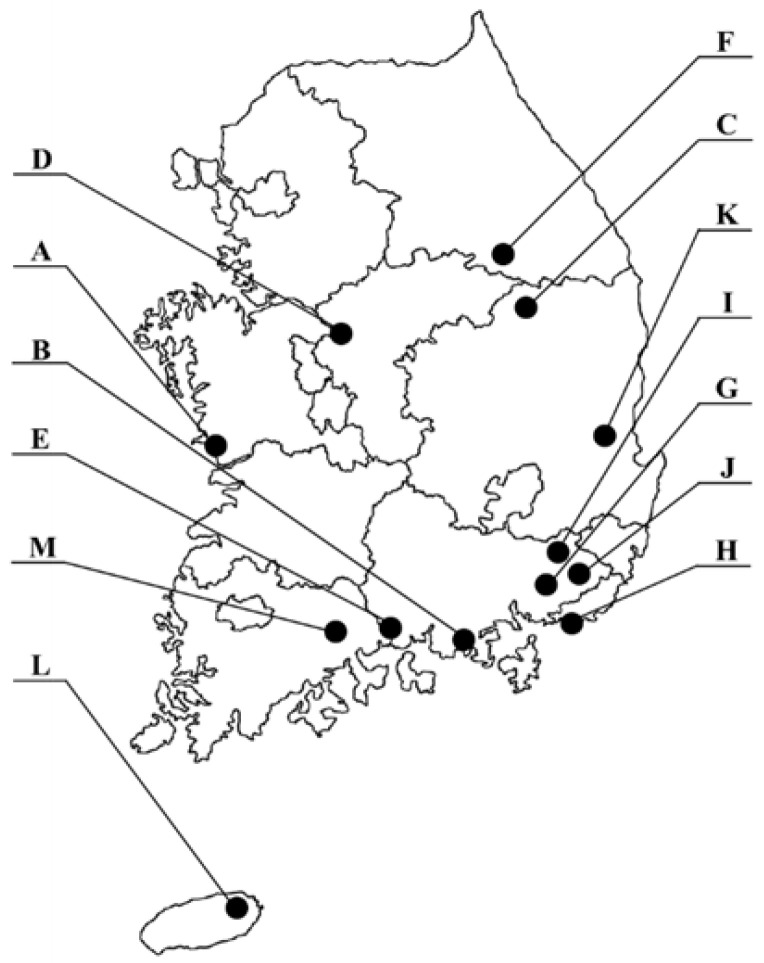	**4th KoNEHS**	General Population (Adult)	2984	1.58 (1.56, 1.61)	3.11 (3.04, 3.19)	0.49 (0.47, 0.50)
	**FROM study**	Total	1157	1.76 (1.72, 1.84)	3.24 (3.13, 3.37)	1.34 (1.27, 1.41)
		A	122	2.04 (1.85, 2.45)	2.39 (2.14, 2.66)	1.60 (1.37, 1.86)
		B	142	2.19 (2.03, 2.36)	4.61 (4.15, 5.11)	2.66 (2.34, 3.01)
		C	121	3.71 (3.33, 4.13)	3.06 (2.76, 3.42)	1.83 (1.56, 2.13)
		D	55	1.54 (1.40, 1.68)	1.80 (1.56, 2.08)	0.84 (0.67, 1.05)
		E	113	1.71 (1.59, 1.85)	4.62 (4.26, 5.05)	1.25 (1.08, 1.43)
		F	92	1.89 (1.67, 2.16)	1.91 (1.67, 2.17)	0.92 (0.76, 1.10)
		G	68	1.47 (1.32, 1.63)	3.30 (2.86, 3.78)	0.94 (0.80, 1.11)
		H	240	1.47 (1.39, 1.54)	3.63 (3.37, 3.89)	1.08 (0.97, 1.19)
		I	35	1.18 (1.00, 1.40)	2.67 (2.27, 3.12)	1.11 (0.86, 1.44)
		J	42	1.03 (0.89, 1.22)	3.03 (2.60, 3.58)	1.83 (1.49, 2.26)
		K	30	1.63 (1.42, 1.86)	4.03 (3.20, 5.02)	0.81 (0.62, 1.06)
		L	47	1.32 (1.19, 1.47)	5.71 (4.89, 6.64)	1.33 (1.08, 1.64)
		M	50	1.28 (1.11, 1.45)	2.38 (2.08, 2.72)	1.29 (1.08, 1.51)

[Note] The figure on the left: an indication of the survey area (13 sites). Pb: lead; Hg: mercury; Cd: cadmium; GM: geometric mean; CI: confidence interval.

**Table 2:** The distribution of the integrated concentrations of three heavy metals.

			Σ HM					
		N	AM ± SD	GM (95% CI)	Min	Median	p95	Max
**4th KoNEHS**	General Population (Adult)	2984	6.53 ± 4.39	5.70 (5.60, 5.80)	1.13	5.47	13.18	93.62
**FROM study**	Total	1157	8.09 ± 4.34	7.24 (7.06, 7.43)	1.81	7.12	15.80	62.64
	A	122	7.60 ± 3.63	6.87 (6.35, 7.45)	2.51	6.44	15.79	19.67
	B	142	11.45 ± 4.74	10.61 (9.90, 11.34)	3.51	10.52	20.93	30.15
	C	121	10.83 ± 5.08	9.84 (9.10, 10.65)	3.20	9.58	20.16	31.78
	D	55	4.92 ± 2.00	4.59 (4.18, 5.08)	2.63	4.48	8.19	13.29
	E	113	8.65 ± 2.99	8.19 (7.73, 8.70)	3.67	8.07	14.97	20.26
	F	92	6.30 ± 6.40	5.41 (4.96, 5.96)	2.32	5.03	10.93	62.64
	G	68	6.69 ± 2.48	6.27 (5.77, 6.81)	2.99	6.09	11.49	12.71
	H	240	7.30 ± 3.18	6.74 (6.41, 7.09)	1.81	6.68	12.91	24.28
	I	35	5.82 ± 2.14	5.48 (4.82, 6.12)	2.48	5.28	11.00	11.71
	J	42	6.94 ± 3.29	6.39 (5.69, 7.30)	3.25	5.72	12.94	21.27
	K	30	7.59 ± 2.67	7.16 (6.36, 8.15)	3.43	7.24	12.90	13.40
	L	47	9.81 ± 4.31	9.00 (8.03, 10.25)	3.59	8.87	19.68	23.42
	M	50	5.61 ± 1.63	5.37 (4.93, 5.84)	2.71	5.51	8.67	9.78

[Abbreviation] AM: arithmetic mean; SD: standard deviation; GM: geometric mean; CI: confidence interval. Σ HM: the integrated heavy metals of blood lead, blood mercury, and urine cadmium.

**Table 3:** Odds ratios for each heavy metal and integrated heavy metal concentrations in relation to eGFR decline.

		eGFR <60 mL/min/1.73 m^2^					eGFR <90 mL/min/1.73 m^2^			
			β	SE	*p*-Value	OR (95% CI)	β	SE	*p*-Value	OR (95% CI)
**4th KoNEHS** **(Total)**	Model 1	BPb	0.092	0.126	0.469	1.096 (0.856, 1.404)	−0.027	0.053	0.611	0.973 (0.877, 1.080)
	Model 2	BHg	−0.067	0.052	0.197	0.936 (0.846, 1.035)	−0.023	0.012	0.049	0.977 (0.955, 1.000)
	Model 3	UCd	0.182	0.077	0.018	1.199 (1.031, 1.395)	0.177	0.060	0.003	1.194 (1.061, 1.344)
	Model 4	BPb	0.113	0.126	0.370	1.120 (0.874, 1.434)	−0.019	0.053	0.727	0.982 (0.885, 1.089)
		BHg	−0.069	0.052	0.188	0.933 (0.843, 1.034)	−0.024	0.012	0.047	0.976 (0.954, 1.000)
		UCd	0.178	0.079	0.025	1.194 (1.022, 1.395)	0.181	0.061	0.003	1.199 (1.064, 1.350)
	Model 5	Σ HM	−0.251	0.313	0.421	0.778 (0.421, 1.435)	−0.100	0.105	0.344	0.905 (0.737, 1.112)
**4th KoNEHS** **(Age** **≥ 50)**	Model 1	BPb	−0.101	0.128	0.431	1.106 (0.860, 1.422)	−0.038	0.057	0.502	0.963 (0.861, 1.076)
	Model 2	BHg	−0.063	0.052	0.223	0.939 (0.848, 1.039)	−0.019	0.012	0.107	0.981 (0.958, 1.004)
	Model 3	UCd	0.191	0.079	0.016	1.210 (1.037, 1.413)	0.168	0.063	0.008	1.183 (1.046, 1.338)
	Model 4	BPb	0.123	0.129	0.342	1.131 (0.878, 1.456)	−0.031	0.057	0.589	0.970 (0.867, 1.084)
		BHg	−0.065	0.052	0.214	0.937 (0.845, 1.038)	−0.019	0.012	0.108	0.981 (0.958, 1.004)
		UCd	0.187	0.082	0.022	1.206 (1.028, 1.415)	0.171	0.063	0.007	1.186 (1.048, 1.342)
	Model 5	Σ HM	−0.220	0.318	0.489	0.803 (0.430, 1.496)	−0.063	0.115	0.584	0.939 (0.750, 1.176)
**FROM study**	Model 1	BPb	0.026	0.165	0.875	1.026 (0.742, 1.419)	0.164	0.122	0.180	1.178 (0.927, 1.497)
	Model 2	BHg	−0.603	0.153	<0.001	0.547 (0.405, 0.739)	−0.495	0.112	<0.001	0.610 (0.490, 0.760)
	Model 3	UCd	−0.266	0.105	0.012	0.767 (0.624, 0.943)	−0.179	0.078	0.022	0.836 (0.717, 0.974)
	Model 4	BPb	0.109	0.169	0.522	1.115 (0.800, 1.553)	0.232	0.125	0.064	1.261 (0.986, 1.612)
		BHg	−0.580	0.154	<0.001	0.560 (0.414, 0.757)	−0.485	0.113	<0.001	0.616 (0.493, 0.768)
		UCd	−0.245	0.108	0.023	0.783 (0.634, 0.967)	−0.173	0.080	0.030	0.841 (0.719, 0.984)
	Model 5	Σ HM	−0.567	0.212	0.007	0.567 (0.375, 0.859)	−0.483	0.151	0.001	0.617 (0.459, 0.829)

[Abbreviation] eGFR: estimated GFR; β: logistic regression coefficient; SE: standard error; OR: odds ratio; CI: confidence interval; BPb: lead in blood; BHg: mercury in blood; UCd: cadmium in urine. Σ HM: integrated heavy metal concentration (BPb, BHg, UCd). [Note] All models are adjusted by age, gender (reference value: female), and body mass index (BMI).

## Data Availability

The data presented in this study are available on request from the corresponding author. The data are not publicly available due to the lack of ethical approval for the public release of personal research data.

## Supplementary Materials

The following supporting information can be downloaded at https://www.mdpi.com/article/10.3390/diagnostics15010086/s1: Table S1. The number of study participants in the environmentally vulnerable area (FROM study).
