# Supplementary material for: A Comparative Study on the Paradoxical Relationship Between Heavy Metal Exposure and Kidney Function
Source: Diagnostics (Basel). 2025 Jan 2;15(1):86. doi: 10.3390/diagnostics15010086 (PMC11719648; doi:10.3390/diagnostics15010086)
Supplement: Supplementary file 1 [file diagnostics-15-00086-s001.zip › diagnostics-3343001-supplementary.pdf]

Supplementary Tables List

Table S1. The number of study participants in the environmentally vulnerable area (FROM study).

Table S1. The number of study participants in the environmentally vulnerable area (FROM study). (Unit: N)

		KoNEHS (Reference)	FROM (Environmentally Vulnerable Area)												
			**A**	**B**	**C**	**D**	**E**	**F**	**G**	**H**	**I**	**J**	**K**	**L**	**M**
**Total**	N	2984	122	142	121	55	113	92	68	240	35	42	30	47	50
**Gender**	Male	1296	43	55	47	19	38	50	24	58	11	18	12	22	14
	Female	1688	79	87	74	36	75	42	44	182	24	24	18	25	36
**Age (years)**	<40	629	0	3	1	1	1	0	1	1	0	0	0	0	0
	40-<50	572	0	7	2	1	6	0	0	5	0	0	1	5	0
	50-<60	662	9	17	12	5	10	0	4	33	1	2	2	18	4
	60-<70	732	33	34	36	19	31	9	28	107	15	20	9	22	12
	≥70	389	80	81	70	29	65	83	35	94	19	20	18	2	34
**eGFR (ml/min per 1.73 m^2)**	<60	48	10	7	24	4	11	43	11	16	5	4	2	1	7
	60-<90	161	61	41	62	35	46	40	37	68	20	13	15	10	24
	≥90	2319	51	94	35	16	56	9	20	156	10	25	13	36	19
